# Smart Metering Cybersecurity—Requirements, Methodology, and Testing

**DOI:** 10.3390/s23084043

**Published:** 2023-04-17

**Authors:** David Kohout, Tomas Lieskovan, Petr Mlynek

**Affiliations:** Department of Telecommunications, Brno University of Technology, Technicka 12, 61600 Brno, Czech Republic

**Keywords:** methodology, DLMS, smart metering, cybersecurity, AMM

## Abstract

This paper addresses the current challenges in cybersecurity of smart metering infrastructure, specifically in relation to the Czech Decree 359/2020 and the DLMS security suite (device language message specification). The authors present a novel testing methodology for verifying cybersecurity requirements, motivated by the need to comply with European directives and legal requirements of the Czech authority. The methodology encompasses testing cybersecurity parameters of smart meters and related infrastructure, as well as evaluating wireless communication technologies in the context of cybersecurity requirements. The article contributes by summarizing the cybersecurity requirements, creating a testing methodology, and evaluating a real smart meter, using the proposed approach. The authors conclude by presenting a methodology that can be replicated and tools that can be used to test smart meters and the related infrastructure. This paper aims to propose a more effective solution and takes a significant step towards improving the cybersecurity of smart metering technologies.

## 1. Introduction

Smart metering refers to the method of measuring consumption-based commodities, such as electricity, water, gas, and heat. This paper is mainly focused on testing the security of electricity smart meters (SMs). Smart metering can be used in combination with different protocols and standards, but the most widely used standard [1] is called DLMS (device language message specification).

In the Czech Republic, distribution companies must deploy smart meters for customers by 2027, if the annual consumption at a particular point exceeds 6 MWh. This means deploying not only smart meters, but also a completely new related supporting infrastructure, e.g., a communication infrastructure and a data center (head end system) with a cryptographic key management system. This is because the existing systems and infrastructure are not ready for smart metering deployment, with cybersecurity requirements and real-time reading capabilities.

The main motivation for creating security requirements for AMM (automatic meter management) elements in the Czech Decree 359/2020 [2] was the need to specify the minimum acceptable level of cybersecurity of the AMM system, which will be clearly defined and verifiable in practice. Another reason for the definition of AMM requirements, is the fulfillment of the requirements of the European directives (especially the NIS directive [3]) and the legal requirements of the Czech authority in the field of cybersecurity, NÚKIB (National Office for Cyber and Information Security in Czech Republic), which are listed in the act on cybersecurity [4], containing general and organizational requirements at the state level. The decree on cybersecurity contains mainly organizational and technical requirements for individual entities, and recommendations in the field of cryptographic means, containing recommendations of algorithms and parameters of cryptographic systems.

Another reason, is the existence of similar requirements for different regions (e.g., Great Britain, the USA, and The Netherlands), while for the Central European region, and especially for the Czech Republic, a set of specific safety requirements aimed at AMM was completely missing, which was changed only by Annex 4 of Decree [2]. In Annex 4, the minimum cryptographic and technical requirements for the AMM solution are defined and the conditions for the deployment of smart meters are thus clearly defined. So, manufacturers of smart meters and related infrastructure have sufficient knowledge in advance, of the requirements that can be expected in tenders for the roll-out of smart meters in the Czech Republic. The aim of the article, is to summarize the requirements for cybersecurity, show their importance, and at the same time show the necessity of testing the requirements from Annex 4 [2] before the deployment (roll-out).

Another important consideration, is the need to assess wireless communication technologies depending on the cybersecurity requirements. To this end, it is crucial to develop methodologies for testing security requirements and designing tools and means for their evaluation. The contribution of this article is threefold. Firstly, it summarizes the requirements for cybersecurity, shows their importance, and at the same time shows the necessity of testing the requirements before the roll-out. Secondly, the methodology for verifying the cybersecurity parameters of smart meters and related infrastructure is designed and investigated. Thirdly, a real smart meter is evaluated according to the proposed methodology, and at the same time, the considered wireless communication technologies are tested in the context of cybersecurity requirements.

### 1.1. Structure of the Paper

To provide a comprehensive understanding of the topic, this article is divided into several sections. Section 1 presents related work, summarizes the legislation regarding smart grids, and defines the motivation and goals of this work. Section 2 summarizes the security requirements from the Czech legislation and also describes basic principles and the security side of the DLMS standard. Section 3 is dedicated to the design of the security requirements for AMM. Section 4 describes our testing methodology for testing SMs. It also contains some of our results from testing and also multiple insights that we discovered from our experience with testing SMs and with cooperation with SM manufacturers. Section 5 describes tools that can be used to test the security of SMs and also compares some security-related tests with the Decree [2]. Our testing regarding the cybersecurity of SMs, using wireless cellular technologies, is outlined in Section 6.

In Section 7, this paper extends the solution defined by the ENCS (European Network for Cyber Security) and provides a contribution in the form of a defined set of tools, procedures, and rules for testing specific security requirements of smart meters. It also includes the design of input prerequisites and DLMS protocol parameters for testing, and takes into consideration the limitations of wireless mobile communication technologies. While Czech legislation sets requirements for cryptographic mechanisms and technical requirements, foreign recommendations, such as those from the UK’s National Cyber Security Centre and Germany’s BSI (Federal Office for Information Security) guidelines, offer different perspectives on AMM models and security requirements.

In Section 8, this article concludes a repeatable methodology, input prerequisites, and tools for testing smart meters and related infrastructure to meet cybersecurity requirements. Future work will focus on optimizing the methodology, updating requirements, and adapting it to other protocols, such as IEC 60870-5-104.

### 1.2. Related Works

The background for the main topic of this article (smart metering cybersecurity), is the current situation, in which distribution companies are required to install new smart meters by 2027. Because the deployment of the new smart meters will be mostly selective (due to the consumption requirement), it is ideal to use wireless technologies in the licensed band. Considered technologies are NB-IoT and LTE Cat M1, according to the specification from 3GPP (3rd Generation Partnership Project). However, one of the major challenges facing the installation of smart meters, are the new requirements for cybersecurity, which have not been considered for previously deployed electricity meters (which may have had unsecured communication or only basic assurance of confidentiality, with fixed keys). This paper aims to address the problem of unclear specifications for security requirements in smart meters, as well as the lack of methodologies, procedures, and tools to verify these requirements.

Table 1 represents the analysis of related works, the column ‘DLMS’, specifies whether the article uses DLMS as a main protocol, and the column ‘Method’, represents the main method used in the given article.

The papers listed above, cover various aspects of smart metering security. Some of the papers focus on the analysis of security risks and attacks on smart metering systems, while others propose solutions to address these security issues. Overall, the papers provide a comprehensive overview of the various aspects of smart metering security.

The first one is a recommendation dealing with the methodology for testing the security of smart meters. The ENCS is a non-profit organization, supporting the deployment of secure European critical energy networks and infrastructure [5]. For smart meters, ENCS has proposed security requirements for smart meters and concentrators, meaning that the requirements apply to the communication from the smart meter to the concentrator and from the concentrator to the central HES (head end system), but not to the security of the HES itself. The requirements are defined by ENCS [5] in general terms for a smart meter and a concentrator/gateway. For each requirement, its security test is listed. The security tests are divided into three categories: (i) documentation assessment; (ii) functional tests; (iii) resistance tests and penetration tests. Therefore, ENCS provides requirements and methodology targeting smart meter security, but does not provide tools and procedures for real testing. At the same time, the setup and configuration requirements, especially for the DLMS protocol, which are necessary for the testing, are not defined. The ENCS methodology is primarily focused on the concentrator/gateway communication concept, using power line communication [25], and does not consider the recent trend of selective smart meter roll-outs with mobile or radio communication (NB-IoT or LTE Cat M [26]).

One of the papers [6], focuses on practical attacks on DLMS, particularly message modification, and the security suite’s problems. The article provides an overview of security in general and recommendations for individual levels’ weaknesses. Another paper [7] addresses basic and advanced key management issues, including cryptographic algorithms and complex distribution schemes, and explores the potential use of physically unclonable functions in smart meters.

Another set of papers assess potential security-related risks that can occur in smart meters and adjacent infrastructure. Reference [8], is based on risk related to DLMS, whereas paper [9] deals with the wider area of the AMI. Another paper [10] examines the security and privacy aspects of smart metering infrastructure. The paper lists potential attackers, security threats, and attacks on smart metering infrastructure, and presents security approaches to address these issues. Reference [11] provides general information about smart metering, including physical security, theft detection, and communication standards.

Reference [12] deals with the design of an intrusion detection system, that can be made more precise with the use of RAM and CPU. Reference [13] examines the possible risk of using correlation between the length of encrypted messages and the overall consumption. This could be taken advantage of by burglars.

Other papers focus on specific security issues related to smart metering, e.g., reference [14] proposes a new authentication scheme for smart metering infrastructure using a quantum communication protocol. Another paper [15] presents a new protocol for creating a secure communication path wirelessly, primarily used in other IoT meters (temperature or humidity sensors), where communication occurs through individual meters. Other references [16,17] propose lightweight protocols for verifying both parties and transferring encryption keys.

Another paper [18] addresses the issue of implementing DLMS communication into the cyber range. This article is not so much focused on security, as it is on teaching and training. The authors have created a virtualized power meter and concentrator, that are dynamically generated using cyber range. As the authors write in the conclusion, security is only the next phase of their research.

In terms of testing, several studies [19,20,21,22,23] propose different methods for testing the reliability and security of smart metering systems. These methods range from validating firmware to testing voltage levels, power outages, and different voltage combinations. All of these tests have an impact on the overall security of these devices. The last reference in Table 1 [24] deals with the fire safety of smart meters.

In conclusion, these papers provide a comprehensive overview of the various aspects of smart metering security, ranging from the analysis of security risks, to proposing solutions to address these issues. The papers demonstrate the importance of ensuring the security and reliability of smart metering systems, given the sensitive data they collect and the potential consequences of a security breach. Therefore, we are working on a system that focuses on requirements, methodology, and testing the cybersecurity of smart meters. We also consider NB-IoT and LTE Cat M1, together with security issues.

### 1.3. Motivation and Goals

Tenders for energy network operators (utility) for smart meters and related infrastructure, are currently being prepared or are already in process in the Czech Republic. Due to legislation, distribution companies in the Czech Republic must install smart meters for customers with an annual consumption exceeding 6 MWh, by 2027. With regard to the direct network connection of smart meters and other devices (e.g., RTU—remote terminal units to control photovoltaic power stations) to important systems related to the control of the distribution system, which is part of the critical infrastructure, it is necessary to define the security requirements and testing methodology for these devices. Related to this, is also the creation of an experimental background that will allow testing the security requirements of newly developed devices and specific protocols (e.g., DLMS/COSEM, IEC 60870-5-104).

According to the analysis of related works, in Section 1.2, the methodology and tools for testing the security requirements of smart meters are currently missing. The main goals of this article are the following:

- introduce security requirements for smart meters and design a methodology to test these requirements;
- provide evaluations and measurements according to the proposed methodology;
- design and verify tools, software, and equipment for evaluation and measurement according to the proposed methodology;
- provide recommendations for smart meter roll-out, based on security requirements considering wireless cellular communication technologies.

## 2. Background

This section describes legislation documents regarding security in smart grids, then some basic concepts regarding the DLMS standard are presented. The last part of this section describes all of the security-related features that are currently specified in the DLMS standard.

### 2.1. Legislation Regarding Smart Grids

The European Union started working on legislation for the deployment of smart meters in 2009, and in this year, three standardization subjects were joined with Mandate M441 [27]. These subjects’ ESOs (European Standardization Organizations), were CEN (Comité Européen de Normalisation), CENELEC (Comité Européen de Normalisation Électrotechnique), and ETSI (European Telecommunications Standards Institute). This joint group was named the Smart Meters Coordination Group (in short SM-CG). Their joint efforts resulted in multiple recommendations and reports, and all documents can be accessed via [28]. The most useful of these documents are probably [29,30,31,32]. Also, there is one book [33] that describes in more detail all the possible threats, vulnerabilities, and cybersecurity standards applicable to the electricity sector, and also discusses the costs of cybersecurity management.

Other documents, from multiple different organizations, also appeared, e.g., the non-profit organization ENCS in cooperation with ENISA (European Union Agency for Cybersecurity), released a paper about security requirements for procuring smart meters [5]. In 2014, there was a report from CERRE (Centre on Regulation in Europe), about the regulation of smart meters in Europe, with multiple challenges [34]. The EU also made multiple legislation frameworks about cybersecurity in critical infrastructure [35]. All these frameworks should be applied to the security of smart meters, because they are a part of the critical infrastructure. In the Czech Republic, the legislation specifies security requirements in Annex 4 of Decree 359/2020 Coll. [2], see more in Section 3.

### 2.2. DLMS Basics

The DLMS standard is very complex. To better understand how DLMS is used, this section describes the basic principles of communication in DLMS. It defines basic principles on OSI/ISO layers 4–7. It is not dependent on any specific communication technology, so it can be used in combination with wireless (e.g., LTE, NB-IoT, wireless M-Bus, GPRS) or wired (e.g., RS-485, Ethernet, M-Bus) technologies. The DLMS standard can be divided into two main parts. The first one is responsible for the communication, providing security features and the base messaging (all described in the DLMS “Green Book” [36]). The second part is called COSEM (companion specification for energy metering) and OBIS (object identification system), and is responsible for storing measured values, object modeling, and also providing configuration options (described in the DLMS “Blue Book” [37]).

Smart meters which use DLMS contain multiple objects, that can be remotely accessed. These objects contain all possible measured values, such as instantaneous voltage, current, and consumption. Then, there are profile objects, that contain the history of all measured values on all phases. Smart meters can also be responsible for billing. The last main set of objects is used for configuration of communication, security, and access levels.

Table 2 presents an example DLMS object of the register type, which is the most common for measured values. The first attribute (logical name) is the pointer or address to the object, and this particular object contains information about the instantaneous voltage on phase 1. The second attribute (for the register object) contains the measured value, and the last attribute contains the unit of the value (in this case, voltage) and scaler. The scaler is an integer value that represents the exponent of base 10 and is used to shift the decimal point of the value. By combining the value (2453), scaler (–1), and unit (V), the reading of this object results in a value of 245.3 V.

### 2.3. Security in DLMS

The DLMS standard includes several security-related sections, to ensure authentication and data protection. The authentication takes place during the first phase of communication, in which the client must authorize themselves to the server before accessing any objects. DLMS currently supports eight authentication levels, which can be divided into three sections (the first two of which contain only one authentication method each).

The first authentication level (no security) should only be used for public access, because it provides no authentication. The second authentication mechanism is called LLS (low-level security), which uses a pre-shared secret key that is sent in an open form and can be captured and maliciously reused. Neither of these methods can ensure secure authentication on remote connections.

The last section is called HLS (high-level security), and it uses the challenge–response model, which requires four messages to be exchanged between the two sides to successfully connect to the meter. HLS can be combined with other algorithms to securely respond to challenges [38]. The supported algorithms are GMAC (Galois Message Authentication Code), SHA-256 (secure hash algorithm), and EC-DSA (elliptic curve digital signature algorithm); no longer secure are MD5 (message digest 5), and SHA-1. When using EC-DSA, neither party needs to know any pre-shared secret. The authentication is based on the digital signature using known certificates and on the PKI infrastructure (root authority) that signed the certificates for the parties (that both sides use to authenticate themselves).

In DLMS, data protection is defined in multiple security levels, called security suites (see Table 3). The AES (Advanced Encryption Standard) is currently specified in DLMS for encryption in all available suites, in combination with the GCM (Galois Counter Mode). The first security suite 0 (in short SS0), uses a pre-shared key for encryption, whereas SS1 and SS2 assume the use of key exchange algorithms to establish symmetric keys for encryption. The DLMS standard supports multiple key exchange models, which are specified by NIST (National Institute of Standards and Technology) [39] and all models are using the ECDH (elliptic curve Diffie–Hellman) key exchange algorithm [38].

### 2.4. Concerns Regarding Security Suite

From the security point of view, the majority of security requirements in the area of AMM and smart meters are known primarily in the context of the DLMS security suite (Table 3). Most currently installed and operated smart meters are at most using SS0, where confidentiality and integrity is ensured, and the encryption keys are exchanged via the AES-128 key wrap method.

However, solutions built on SS0 are no longer secure in the long term, due to the key sizes. NIST [40], ECRYPT [41], ENISA [42], and NÚKIB [43] all recommend using the AES-256 after the year 2030 (recommended algorithms are described further in the following section, in Table 4). There are also multiple security risks and problems related to the short key sizes used in SS0 and also the absence of key agreement algorithms:

1. disclosure of used keys, that results in a complete security breach (including past, present, and future communication);
2. forgery of compromised keys;
3. replay attacks, brute force attacks, and dictionary attacks;
4. risks related to the need of securely storing all keys for all meters (in utility systems);
5. risks associated with the impossibility of centrally and quickly changing keys;
6. risks associated with manual device configuration and the need to pre-load or individualize static keys by staff after deployment of the electricity meter.

The risk described in point 5 can be mitigated with the use of key exchange algorithms. That is why the SS1 and SS2 should use asymmetric cryptography, mainly the ECDH algorithm. According to NIST [44], if the symmetric master key (key for exchanging new symmetric keys using the key wrap method) is compromised, it should be replaced, and the new key must be independent from the compromised key. This cannot be fulfilled with the symmetric key exchange that is supported in DLMS. When using asymmetric key exchange algorithms (the only one supported in DLMS is ECDH), this independence is no longer an issue. In addition, all the keys (including the master key) need to be periodically changed, to fulfill the cryptoperiod recommendations from NIST [40].

When we take into consideration the expected life span of an electricity meter (over 15 years), we need to use the future security recommendations, and that means using only SS2, but for the next few years, even SS1 is sufficient (until 2030).

## 3. Design of Security Requirements

The design of security requirements for smart meters is critical for ensuring the security and reliability of these systems. Smart meters, and the wider smart grid infrastructure, are vulnerable to a range of cyber threats, such as unauthorized access, denial-of-service attacks, and data breaches. The design of security requirements for smart meters and smart grids should address these vulnerabilities and include measures such as secure communication protocols, strong authentication mechanisms, and real-time threat detection and response capabilities. Additionally, the security requirements should also consider the privacy of energy consumption data collected by smart meters. A comprehensive design of security requirements can help to ensure that smart meter and smart grid technology can operate safely and securely, in a rapidly evolving threat landscape.

Table 4 lists multiple cryptographic algorithms and protocols (including their assumed lifetime). These protocols are mainly based on requirements from NÚKIB, ENISA, and NIST. Based on this table, the minimal cryptographic requirements were created, and now are defined in Annex 4 of Decree 359/2020 [2]. This table also shows the compliance with the requirements from [2] and with the DLMS security suite, where the DLMS security suite is not considered to be secure after 2036.

Table 4 defines minimal security levels for the individual periods (“By 2030”, “2030–2036”, “Past 2036”). These time periods are not periods where the SMs are tendered or purchased but in which SMs will be installed and in operation. When we take into consideration that an electricity meter has a life span around 20+ years, we need to assume that all the SMs will have to meet the highest level of cryptographic security in the second half of their life span. The SM does not have to support the higher security level (“over 2036”) from the beginning (at the time of installation) but the new security algorithms can be updated in the SM later. This is also the technical requirement no. 15: “Remote update of security features and cryptographic primitives” (more technical requirements from [2] are described in Table 5).

## 4. Testing Smart Meters

The industry of smart metering has been growing rapidly, and it has become increasingly important to ensure the security and reliability of smart meter devices. However, there has been a lack of a standardized testing methodology and tools for this purpose. While organizations such as the ENCS have developed requirements and methodologies for smart metering, there is no widely accepted testing environment that is publicly available.

In response to this need, a new testing methodology has been developed, that is based on the standards set by NIST and ENISA. This methodology provides a standardized framework for testing the security and reliability of smart meter devices, and it is designed to be used in conjunction with existing testing tools and environments. By adopting this methodology, smart meter manufacturers and utilities can ensure that their devices meet the highest standards of security and reliability, which is crucial for maintaining public trust in the smart grid.

The introduction and the comparison of related work, revealed that there is not enough articles about testing the security of smart meters, or the security testing is not described there in sufficient detail. Therefore, this section is dedicated to introducing our testing methodology and also to presenting some interesting results and insights from our experience with the testing.

### 4.1. Testing Methodology—Basics

The main part of our work was the development of a new testing methodology, to test the cybersecurity of SMs for deployment in the Czech Republic. Moreover, our methodology and the related testing, can be used to test any smart metering device for deployment in any country, even though the methodology is based on a specific Czech law [2]. Thanks to the cooperation with electricity distribution companies (utilities) and smart meter manufacturers in the National Action Plan for Smart Grids in the Czech Republic, the security requirements and the methodology were verified and discussed together [45]. In 2022, the methodology was applied to the testing of smart meters from a foreign manufacturer, and to the testing of smart meters that will be used for the roll-out [46,47]. For the foreign manufacturer, the evaluation was performed to check the security issues for Czech requirements, i.e., compliance with Annex 4 of Decree 359/2020 [2].

Our methodology defines three possible test levels for every test. When a test defines multiple levels, all levels are stacked on top of each other. For example, when a test uses level 3, all other levels need to be verified as well. These levels are:

1. Documentation: security requirement is verified according to documentation from the manufacturer.
2. Functional test: verified with normal operational procedure.
3. Security test: verified with security tests (e.g., attack, wiretapping).

The methodology is available for manufacturers before testing, it describes every test with the testing steps, assessed parameters, and also the final evaluation. The test definition is followed by the test result, testing description (what we did or what we found), and the last part is a recommendation for some small adjustments or some test insights that are important to mention. The test can have three different outcomes, which are described in Table 6.

### 4.2. Testing Requirements

From the security requirements described in Section 1 and Section 3, we developed a set of tests, that are part of our methodology. The most critical security requirements are displayed in Table 7, organized into several sections, each containing a set of tests based around a common security-related group. Each row in the table represents a specific test and includes a unique test ID (using the letter M and a number, e.g., M1), a brief description of the test, and a column describing the tool used to verify the security part of the test. This last column corresponds to one or many tools that are described further in the next Section 5. Some tests can be verified with only one tool, but most tests need a combination of tools to accurately verify the security requirement fulfillment. In the case of the cell being empty, the test requirement can be verified either through a functional test, utilizing regular operational procedures, or by relying on the assertions made in the manufacturer’s documentation.

### 4.3. Insights from Testing

One of the most problematic requirements, is the non-fulfillment of the minimal cryptographic requirements from [2] (test M30). The main failure of this is the missing key management using key agreement algorithms (asymmetric cryptography using ECDH). Consequently, each device must be injected with a unique master key during the manufacturing process. However, if the master key were to be compromised, it would pose a significant challenge, since it cannot be changed remotely. Therefore, changing the master key in the future would require physical access to the device. All risks related to the master key for key wrapping exchange, are already described in Section 2.4.

From the testing perspective, there is a need for remote update of the security functionalities (test M27 and M29) and cryptographic primitives (technical requirement no. 15 from [2], described in Table 5). To fully fulfill this, we also need to test the sufficient future hardware resources (test M28) of the tested devices (this relates to [48]). We can achieve this using the following procedure:

1. The evaluator shall verify, according to the documentation of the device, these entries:
  - the available RAM and flash of the device and compare them to the actual usage while in normal operation, according to the functional specification;
  - the available computing power and the required minimal computing power for the normal operation, according to the functional specification.
2. The evaluator shall verify, based on the documentation and verification of the actual state, that the device has available computing power, RAM, and flash memory for running all security algorithms (Table 3). The evaluator shall also verify, from the documentation, that the device has the computing power, RAM, and flash memory for the increased demand on resources for the future cryptographic algorithms, by at least 100%. To meet the requirement of accommodating 100% more resources, twice the number of resources utilized during an average month of normal operation at the time of deployment (this condition assumes that the device is utilizing at least DLMS SS1), the device must primarily allocate additional resources on the communication side, where the cryptographic algorithms are implemented.

This requirement has to be verified in close cooperation with the manufacturer. From our experience, it is very problematic, and this requirement is usually not fulfilled (e.g., the device is using DLMS SS0 and the memory and computational resources are already using over 50% of the available resources).

The testing process has uncovered an additional insight, indicating that the manufacturer often disregards the importance of documentation, which is critical for the testing. Multiple documents are frequently supplied, which may be inconsistent, leading to difficulty in locating the relevant information required for testing. While documentation is a vital element in conducting a comprehensive test, the documentation provided by the manufacturer is often imprecise and fails to cover all the necessary requirements.

### 4.4. Designing Initial Prerequisites for Testing Smart Meters

Based on our experience from the testing of smart meters, we have created a set of prerequisites, of essential information that we need to have, to accurately test the whole methodology (Section 4) on supplied devices. In addition, for some tests we need cooperation with the electricity distributor or with the telecommunication company (that the distributor uses for communication). The initial prerequisites for DLMS testing should contain (from our experience with real testing) the following list of information:

1. Basic DLMS parameters for devices:
  - referencing used, LN (logical names) or SN (short names);
  - addressing used on every interface (HDLC or wrapper);
  - all interfaces on the device, their connection and state (e.g., pinout on RS-485);
  - all addresses (DLMS uses client and server addresses);
  - access roles and authentication used (passwords and relation to client addresses);
  - encryption keys and certificates for every access role (when keys cannot be shared directly, we need information about the mechanism, that we can use to obtain the keys by ourselves).
2. Multiple firmware (FW) versions for the testing devices:
  - at least one new FW version, that can be used to finish all security tests (version that is above all other supplied FW versions);
  - at least two FWs with valid signature and correct incremented versions. These two FWs can be alternately applied to the device. If the device cannot apply an older version of FW, we need at least five valid FWs with valid signatures and with incremented versions.
3. Access to the planned central metering point—HES (head end system):
  - having sufficient access permissions to perform all security-related operations on the tested devices (changing keys, passwords, certificates, security setup, etc.);
  - access for capturing all the communication between meters and the HES (to verify used communication and properly applied encryption on the messages). This can be achieved in multiple ways:
    - capturing the communication directly on the device where the HES is;
    - inserting a virtual machine into the infrastructure, such that it can see all the communication (that is heading to the HES).

## 5. Smart Meter Testing—Tools and Procedure

Testing the security of smart meters needs a lot of devices, tools, and applications to be able to test all of the possible security requirements that we specified in the testing methodology.

### 5.1. Testing Procedure

**Figure 1:**
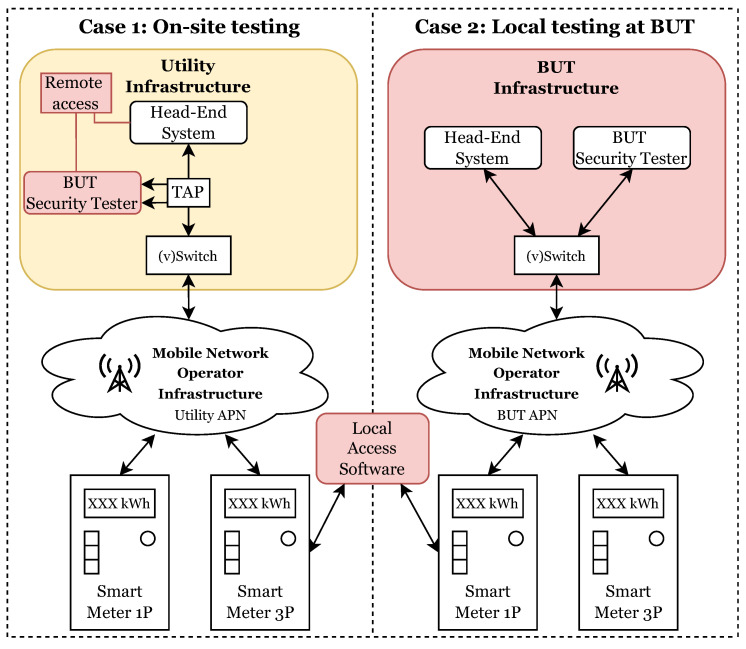
Testing case scenarios, difference between on-site and local testing.

This section focuses on the testing procedures used to evaluate the cybersecurity of smart meters. As can be seen in Figure 1, two cases are discussed: on-site testing and local testing at the Brno University of Technology (BUT). In both cases, a testing methodology was followed, including vulnerability scanning, penetration testing, and analysis of network traffic.

Conducting on-site testing poses significant challenges in terms of access. Access to a distributor’s critical infrastructure typically requires extensive authentication procedures, and network access is often severely restricted, resulting in slow and limited testing. Nevertheless, this approach remains a viable option, as it allows for the concurrent testing of the target solution and its associated infrastructure, which will be deployed later.

Alternatively, local testing offers greater flexibility for smart meter testing, as all communication and testing is conducted under the tester’s control. This approach is considerably faster, as the tester’s mobile network is utilized for testing, instead of the distributor’s network. However, the drawback of this testing method is the inability to test the device’s performance relative to the infrastructure with which it will be deployed to the customer. The whole testing procedure can be performed in a few steps:

1. Research specific smart meter technology: Before selecting tools, it is very important to understand the underlying technology, such as communication protocols (DLMS, ModBus, etc.), types of data (IP, HDLC, etc.), interfaces (P1, optical, serial, etc.), and security mechanisms (SS0, SS1, SS2).
2. Identify testing objectives: It is very important to determine what to test. Different manufacturers implement communication, security, or functions in different ways. Therefore, it is necessary to find possible potent threats.
3. Research available testing tools: Some tools and methods are the same for most tests (e.g., Wireshark), other tools and methods depend on the specific test. It is necessary to find and use the most appropriate tool to obtain the most accurate results.
4. Create a testing plan: Many tests use similar tools and work with the same data. To avoid performing the same steps for multiple tests multiple times, creating a suitable plan can save time and money.
5. Conduct the testing: Perform testing based on the plan. It is necessary to note the results of each test in detail, and document any problems.
6. Evaluate the results: Analyze data collected during testing to identify any threats or problems. This information is important to make recommendations to improve SM technology or to select additional tools for further testing.
7. Repeat the process: As the state of science and knowledge evolves, new testing methods become available. It is highly recommended to perform SM testing on a regular basis (the frequency of security testing can vary depending on various factors such as manufacturer’s recommendations, regulatory requirements, or the internal norms of the company that operates these meters), to ensure that the most effective tools are being used to test the SMs.

### 5.2. Testing Tools

There follows a list of the most commonly used tools to test the security of smart meters, with a description and their benefits for testing. Each smart meter test is unique and therefore each technology is treated individually.

- Kali Linux is a popular Linux-based operating system designed for digital forensics, penetration testing, and cybersecurity auditing. The tools below this point are a standard part of the distribution.
  - arpspoof—an open-source network auditing tool, that allows network traffic to be intercepted and redirected, making it a useful tool for network administrators and security professionals. By spoofing ARP (address resolution protocol) messages, arpspoof can trick devices on a network into sending traffic to a different destination, enabling users to perform man-in-the-middle attacks and capture sensitive information. (In some versions of Kali Linux it might be necessary to install this tool separately.)
  - GreenBone—an open-source tool designed for vulnerability assessment of networked devices, and is particularly useful for identifying security vulnerabilities in IT networks, including those in smart grid infrastructure. (In some versions of Kali Linux it might be necessary to install this tool separately.)
  - hping3—a versatile open-source command-line utility, that allows users to perform advanced network testing and troubleshooting, and performance analysis, including packet crafting, testing firewall rules, and flood attacks.
  - Nmap—a commonly used open-source tool for network mapping, with the ability to detect open ports and vulnerabilities, port scanning, and penetration testing, providing valuable information for securing and managing network infrastructure. In the case of smart meters, it is a useful tool for finding open smart meter ports and estimating the services used on these ports.
  - tcpreplay—an open-source software tool designed for replaying network traffic from files, making it an ideal solution for testing and troubleshooting network equipment and applications. The tool allows already captured traffic to be scanned, and can test if the device is resistant to double processing of one message. Furthermore, the tool can be used to send modified traffic back to the network and test protection mechanisms such as checksum, fuzzy data resilience, or proper TCP communication sequence number checking.
  - Wireshark—a powerful open-source network protocol analyzer that allows users to capture and inspect network traffic in real-time, making it an essential tool for network troubleshooting and optimization.
- Avalanche is a hardware tester, specially designed for emulating clients and servers. It is designed to act as an end device on both sides of communication for testing devices, firewalls, and rules, and is capable of making up to 20 Gbps traffic throughput.
- DATEL is a custom made DLMS client/server application made specially for DLMS security testing. More about this tool is described in the following paragraph. (DATEL is currently not publicly available. However, its functionalities can be implemented using Gurux [49].)
- Frequency analyzer is needed if there is a reasonable suspicion that the device is transmitting data on frequencies and protocols not described in the documentation. The frequency analyzer makes it possible to detect these communications.

To be able to test most of our requirements in the methodology, we needed a tool for testing DLMS-related requirements. One option concerning DLMS-related requirements, was to use the DLMS CTT (conformance test tool), but this tool is very expensive, it does not concern all security requirements defined in Table 7, and it does not provide the flexibility that we needed for our testing. Another option, was to use and modify other existing tools and libraries that implement the DLMS standard. Based on the Gurux’s DLMS library [49], we designed an application for testing smart meters using DLMS.

This application is called DATEL (DLMS application for testing energy labs), it is described in more detail in a standalone article [50]. We are using DATEL as the main tool for testing our methodology, but it can also be used as a manual DLMS client to read any values, it can also be used to test reading different objects that should not be accessible with a lower access level.

The main benefits of DATEL, concerning the security requirements defined in Table 7, are listed below. All points can be linked to specific tests, with the test ID in brackets:

- perform firmware update (M3, M4);
- fuzzy testing (M5);
- check if all unnecessary interfaces are disabled (M8);
- generate messages to fill logs on SM (M11);
- check the log sizes, try to delete them (M11);
- perform a replay attack (M15);
- check if data in messages are encrypted (M16);
- check if data in messages have protected integrity (M16);
- check if cryptographic material can be updated (M17);
- verify log entries (M19);
- check if the SM is measuring values during DoS attack (M22);
- verify that lower roles can access security-related objects (M24);
- verify usage of strong authentication (M25);
- verify whether SM blocks roles after unsuccessful attempts (M26);
- perform security based update (M27, M29).
  DATEL can also be used for tests that are not directly related to the testing methodology:
- testing new communication technologies related to smart metering (e.g., Section 6);
- measuring data volumes of normal operation (e.g., to develop new reading schemes);
- measuring connection parameters: delays, timers, and speeds;
- can be used on a connection that is being tunneled via TLS or IPsec.

One of the last benefits of DATEL, is that it is very lightweight. When we compare DATEL with the production environment, HES usually cannot be easily deployed or provided for testing.

## 6. Cybersecurity in the Context of Wireless Cellular Technologies

Tenders for smart electricity meters and related infrastructure are currently being prepared or are already in process. Due to Decree 359/2020 [2], distribution companies must install smart meters for customers with an annual consumption exceeding 6 MWh by 2027, this predisposes selective installations with wireless technologies in a license band. NB-IoT and LTE Cat M technologies, according to the specification of the 3GPP association, are mainly being considered.

The considered deployment of smart meters also includes placement in “indoor” and “deep indoor” environments (approx. 10–15% of consumption points). From the point of view of radio communication, “indoor” is an environment where the radio signal spreads from the outdoor to the indoor environment. From the point of view of attenuation of the radio signal by the environment, the signal strength may decrease by approximately 20 dB. In the case of “deep indoor”, it is a significantly more demanding environment for the penetration (passage) of the radio signal than in the case of the “indoor” environment. From the point of view of radio communication, in the case of deep indoor, it is an environment where the signal strength may be from −120 dBm to −137 dBm (the signal strength in normal or ideal conditions is −100 dBm). Examples of deep indoor environments include, concrete underground spaces, metal covers of electricity meter boxes in underground spaces, or places where there is insufficient radio signal coverage (signal to interference plus noise ratio, SINR < −3). Due to the typical sensitivity of the receiver for 2G and LTE mobile technologies of about −102 dBm, the coverage may not be sufficient, due to the considered attenuation for “indoor” and “deep indoor” scenarios. Therefore, NB-IoT and LTE Cat-M1 technologies are being considered, which offer a receiver sensitivity of up to −130 dBm.

At first glance, it seems that communication technologies have nothing to do with cybersecurity and the security requirements mentioned in the previous sections. Insights from testing smart meters with NB-IoT and LTE Cat M1 [51], in the context of cybersecurity requirements, are presented in the following sections.

### 6.1. Vendor Backdoor

Analysis of the use of the frequency spectrum is important for proper communication from the point of view of NB-IoT and mobile networks, but also from the point of view of cybersecurity, as it is an important tool for monitoring communication and eliminating transmissions outside the required functions (vendor backdoor). For example, the meter may send random messages that are not documented.

### 6.2. Network Registration

As part of the testing of several different smart meters, an incorrect configuration for registration to the mobile network (NB-IoT) was revealed, when after turning off the smart meter and then turning it on, the communication module did not try to reconnect to the last known base station (cell) and it started the procedure for detection (scanning) of available base stations. This procedure was run for all supported communication technologies, e.g., NB-IoT, LTE Cat-M and also EGPRS, see the point above. The result was an increase in the time window required for the registration of the smart meter to the mobile network (NB-IoT), where the time was on the order of several minutes. This does not fulfill the technical requirement no. 1: safe recovery after an error, outage, or failure. Testing took place in radio conditions corresponding to ECL class 0 (enhancement coverage level), when the signal strength level (parameter RSRP—reference signal received power) was in the range of −60 dBm to −105 dBm. In the case of demanding radio conditions (below −120 dBm, i.e., ECL 2), a significant increase in the time required for successful registration can be expected. So it is crucial to reach a point/configuration that only takes a few seconds for the module to successfully register to the network.

### 6.3. Behavior of Smart Meter in the Case of Limited Radio Conditions

Knowledge of the limit parameters of communication, in case of borderline radio conditions, is key, due to the planned remote updating of security functionalities and cryptographic primitives (technical requirement no. 15 in Table 5). It can be observed that, due to deteriorated (boundary) radio conditions, repeated transmission of the message was carried out. Repeated attempts when sending data in the case of ECL 1 or ECL 2, further increase the communication delay. It is necessary to reflect this fact on the intermediate network elements (firewalls) and adjust the timer settings for the delivery of the message on the side of the surveillance system (central).

In the case of an increase in ambient noise (interference), the communication delay increases and the message may not be delivered. RSRP levels lower than −133 dBm were not recorded during the measurements, and thus the transition to ECL 2 only causes the SINR parameter if its value is lower than −3 dB. In the case of switching to ECL 2, however, robust modulation and coding schemes are used. In the most robust case, it is the BPSK (binary phase shift keying), i.e., digital modulation based on shifting the phase of the harmonic carrier by 0° or 180°, depending on the value of the binary modulation signal. This modulation is therefore used in ECL 2, instead of QPSK (quadrature phase shift keying), i.e., digital modulation that uses four-state keying by phase shift, where each state transmits 2 bits simultaneously. At the same time, it is a basic type of quadrature modulation, as its constellation diagram is identical to 4-QAM modulation. Based on the measurements, it can be concluded that, in the case of this location, 1 message out of 20 sent was not delivered.

## 7. Discussion

Based on the analysis of the current state of the art (Section 1.2), the solution defined by ENCS is very similar. The contributions and important extension of this paper are:

- the definition of tools, procedures, and rules for testing particular security requirements for smart meters;
- design of the initial prerequisites and DLMS protocol parameters for the testing;
- consideration of the testing methodology with respect to the specifics and limitations of wireless mobile communication technologies (point-to-point).

Czech legislation for the upcoming roll-out of smart meters, has defined a set of requirements for cryptographic mechanisms, and a set of technical requirements (Annex 4 of Decree 359/2020 on electricity metering [2]). From the perspective of foreign rules, and recommendations of major institutions, the following standards, norms, and recommendations can be considered for AMM in the Czech Republic:

- NCSC: National Cyber Security Centre (United Kingdom)
  - defines a comprehensive national certification for smart meter security;
  - considers a different system architecture (prepaid tariffs, hubs, and concentrators).
- DLMS: DLMS security suite
  - does not define technical requirements, only cryptographic algorithms and keys.
- BSI profile (Germany)
  - In Germany, the AMM model is different and they are considering a smart meter gateway, which must meet the most strict requirements of the BSI guidelines. These requirements could be fulfilled only by the security at higher layers using TLS or IPSec, and with sufficient hardware to meet the technical requirements.

During the testing of smart meters, a variety of tools were used, to ensure the accuracy and reliability of the devices. Some of the main tools utilized were Wireshark, arpspoof, hping3, tcpreplay, Linux Kali, traffic generator avalanche, frequency analyzer, Greenbone, Nmap, and a custom DLMS application—DATEL. These tools provided a range of functionalities, such as traffic analysis, packet manipulation, frequency analysis, vulnerability scanning, and traffic generation. By utilizing this diverse set of tools, the testing team was able to thoroughly evaluate the smart meters and ensure their secure and efficient operation in real-world scenarios.

Our testing software DATEL, is based on modified open-source code from Gurux, which allows anyone to easily customize and modify the code to meet their specific testing needs. This flexibility is especially useful for companies and organizations with unique testing requirements or specialized equipment. Furthermore, by utilizing open-source code, our testing software benefits from the collective contributions of the community, ensuring that the software remains up-to-date and can be continuously improved over time.

## 8. Conclusions

The key point of deploying smart meters and related infrastructure, is the real laboratory testing to meet cybersecurity requirements, by an independent authority, as part of a tender process. These tests must be carried out prior to actual deployment, otherwise there are potential risks (financial losses, breach of legislation or GDPR, damage to the customer, company name, or customer disconnection). This article defines the necessary prerequisites for the testing, and presents the testing method, tools, and methodology. The main contributions of the results in this article, are the repeatable methodology, initial prerequisites, and tools for testing for evaluation of smart meter security. Future work will focus on further testing of smart meters and related infrastructure, to optimize the methodology and update requirements. At the same time, the aim is to adapt the methodology and tools to other protocols, e.g., IEC 60870-5-104, which is used by RTUs for dispatching decentralized resources.

## Figures and Tables

**Table 1:** The state of the art.

No.	Authors	Year	DLMS	Method	Purpose
[5]	ENCS	2019	✗	C ${}^{2}$/L ${}^{5}$/E ${}^{7}$	Security requirements and methodology
[6]	Shanmukesh Pudi et al.	2021	✓	A ${}^{6}$/L ${}^{5}$/E ${}^{7}$	Practical attacks on SMs, modification of messages
[7]	M. S. Abdalzaher et al.	2023	✗	A ${}^{6}$	Key management analysis for SMs
[8]	Norman Luring et al.	2018	✓	A ${}^{6}$	Analysis of weaknesses of the security layer in DLMS
[9]	Ali Ismail Awad et al.	2023	✗	A ${}^{6}$	Assessment of potential security risks in SMs
[10]	Obaid Ur-Rehman et al.	2015	✗	C ${}^{2}$/A ${}^{6}$	Analysis of security issues in SMs and introduction to possible solutions
[11]	David Bačnar et al.	2022	✗	A ${}^{6}$	Security and privacy analysis with intrusion detection system for SMs
[12]	Chih-Che Sum et al.	2021	✗	C ${}^{2}$	Intrusion detection system for SMs
[13]	Marcell Fehér et al.	2020	✓	M ${}^{4}$/L ${}^{5}$	Correlation between the length of messages and consumption
[14]	Kumar Prateek et al.	2023	✗	C ${}^{2}$/M ${}^{4}$/A ${}^{6}$	New authentication scheme for SMs
[15]	Priyanka D. Halle et al.	2022	✗	M ${}^{4}$/A ${}^{6}$	New protocol for creating secure path through sensors
[16]	Seung-Hwan Ju et al.	2018	✓	C ${}^{2}$	Secure key transfer from manufacturer to operator
[17]	Vijay Kumar et al.	2014	✗	C ${}^{2}$	Design of security protocol to secure AMI systems
[18]	Tomas Lieskovan et al.	2022	✓	S ${}^{1}$	Open-source cyber range for DLMS, training purpose
[19]	Liang Xiaobing et al.	2016	✗	E ${}^{7}$/F ${}^{8}$	Cloud based software for testing SMs
[20]	Himanshu Goyal et al.	2021	✓	W ${}^{3}$/L ${}^{5}$	Validation test bench to test reliability of SMs
[21]	Zhang Leping et al.	2019	✗	L ${}^{5}$	Testing the reliability of SMs’ software implementation
[22]	Peter Janiga et al.	2015	✗	L ${}^{5}$/E ${}^{7}$	Testing system for SMs directed on reliability
[23]	Henrique Mendes et al.	2018	✓	L ${}^{5}$	Tool for testing security during power line communication
[24]	Rim Marah et al.	2020	✗	C ${}^{2}$	Threats for smart meters and proposed system for fire safety of SMs

${}^{1}$ Simulation, ${}^{2}$ concept, ${}^{3}$ working, ${}^{4}$ mathematical, ${}^{5}$ laboratory tests, ${}^{6}$ analysis, ${}^{7}$ experiment, ${}^{8}$ field tests.

**Table 2:** Example of DLMS register object.

Attribute	Name	Value	Note
1	Logical Name	1.0.32.7.0.255	Ch. 0 L1 voltage inst. value
2	Value	2453	Value needs to be combined with scaler
3	Scaler and Unit	S: –1, U: 35	Voltage (35)

**Table 3:** DLMS security suites [36].

Suite ID	Authenticated Encryption	Digital Signature	Key Agreement	Hash	Key Transport	Compression
0	AES- GCM-128	-	-	-	AES-128 key wrap	-
1	AES- GCM-128	ECDSA P-256	ECDH P-256	SHA- 256	AES-128 key wrap	V.44
2	AES- GCM-256	ECDSA P-384	ECDH P-384	SHA- 384	AES-256 key wrap	V.44
3–15	Reserved for future updates					

**Table 4:** Requirements for cryptographic algorithms and protocols (✓—approved and recommended; ✗—legacy and not recommended).

Cryptographic Requirements	By 2030	2030–2036	Past 2036
**Ensuring confidentiality**			
Use of block cipher AES-256	✓	✓	✓
Use of block cipher AES-128, AES-192	✓	✗	✗
**Ensuring confidentiality and integrity**			
Use of block cipher mode GCM, CCM	✓	✓	✓
Use of block cipher mode CTR, OFB, CBC, CFB in combination with secure MAC in mode EncryptThenMAC with approved ciphers	✓	✗	✗
**Ensuring integrity**			
Digital signature DSA 15360 and more, EC-DSA-512 and more, RSA 15360 and more	✓	✓	✓
Digital signature DSA 3072, EC-DSA-256, RSA 3072	✓	✓	✗
Hash SHA2-512, SHA3-512	✓	✓	✓
Hash SHA2-256, SHA2-384, SHA3-256, SHA3-384	✓	✓	✗
Mode for integrity protection HMAC, CMAC	✓	✓	✓
**Ensuring key management**			
DH-15360, ECDH-512	✓	✓	✓
DH-3072, ECDH-256	✓	✓	✗
**Random bit generator**			
HMAC_DRBG, Hash_DRBG both for SHA-1, SHA-224, SHA-512/224, SHA-256, SHA-512/256, SHA-384, SHA-512, SHA3-512	✓	✓	✓

**Table 5:** Technical requirements from [2].

No.	Description
1	Safe recovery after an error, outage, or failure
2	Reliable time synchronization
3	Instructions for safe installation, initialization, and operation supplied together with the device
4	Data validation before its use: protection of inputs
5	Flood protection (DoS) using traffic filtering or network segmentation, resource management
6	Interface minimization: deactivation of all unnecessary services, protocols, and physical interfaces
7	Security events must be recorded and reported, the log must be protected against modification and deletion, min. size for 1000 security records
8	Every device must be uniquely identifiable
9	Data in messages must be encrypted
10	Messages must have integrity protection
11	Execution of commands must be confirmed
12	Access to the elements processing sensitive data requires breaking through a security perimeter with a seal
13	Cryptographic credentials must be unique and securely stored for the smart meter, they must not reduce the security of another smart meter after being stolen
14	Separation of measurement and communication functionality
15	Remote update of security features and cryptographic primitives
16	Remote update of cryptographic credentials

**Table 6:** Test results.

Result	Description
**PASS**	This test was successful. The security requirements are fulfilled.
**FAIL**	This test was unsuccessful. The security requirements are not fulfilled.
PASS *	This test was successful. The security requirements are fulfilled, but there is some space for improvement. This is not related to the test outcome, but it could have an impact on the functional or security side of the whole solution.

**Table 7:** Tests in our methodology.

ID	Description	Tools for Testing
**General Requirements**		
M1	Safe recovery after failure	K ${}^{1,}$${}^{6}$
M2	Instructions for safe installation, initialization, and operation	-
**Firmware Protection**		
M3	Install new firmware only after successful check of digital signature	D/K ${}^{1,}$${}^{6}$
**M4**	Firmware integrity check before startup	D
**Interfaces Protection**		
M5	Data validation before use (input protection, fuzzy testing)	D/K ${}^{1}$${}^{,}$${}^{3}$${}^{,}$${}^{5}$${}^{,}$${}^{6}$
M6	Flood protection (DoS) using traffic filtering or network segmentation	A/D/K ${}^{3}$${}^{,}$${}^{5}$${}^{,}$${}^{6}$
M7	Interface minimization—deactivation of all unnecessary services and protocols	D/K ${}^{2}$${}^{,}$${}^{4}$
M8	Deactivation of all physical and logical interfaces (that are not expected for functional requirements)	D/F
M9	Settings protection—configuration changes can only be made in authorized mode	D
**Logging**		
M10	The meter must report security events	D/K ${}^{6}$
**M11**	Security events must be recorded, this log must be protected against modification and deletion, with minimal size of 1000 security entries	D
**Message Protection**		
M12	The security level of the random number generators must be defined (at least 128 bits)	-
M13	The PRNG (pseudo-random number generator) must conform to NIST standards	-
M14	Randomization of required initialization values of cryptographic algorithms	-
M15	Protection against double processing of the same message (replay of messages)	D/K ${}^{1}$${}^{,}$${}^{5}$${}^{,}$${}^{6}$
M16	The data in messages must be encrypted	D/K ${}^{6}$
M17	Messages must have protected integrity	D/K ${}^{6}$
M18	It is possible to update cryptographic material	D
**Physical Protection**		
M19	When an intrusion is attempted, a log entry is created and a warning message is sent	D/K ${}^{1}$${}^{,}$${}^{6}$
M20	Sensitive data processing components are covered by a security perimeter	-
M21	The device perimeter must be sealed	-
**Resistance**		
M22	Separation of measurement and communication functionalities (device must measure all values, even under DoS attack)	A/D/K ${}^{3}$${}^{,}$${}^{6}$
M23	Maintaining security during/after a failure (device cannot reveal any keys or stop securing messages/enforcing access levels)	D ${}^{1}$${}^{,}$${}^{6}$
**Access Control**		
M24	Separating roles, assigning permissions to roles, assigning roles to interfaces	D
M25	User authentication must use strong authentication methods (strong keys and passwords)	D
M26	Device blocks roles/users after unsuccessful authentication attempts	D
**Future Update**		
M27	Remote update of security functionalities and cryptographic primitives	D
M28	Sufficient future resources (RAM, flash, and computational power) to update security and cryptographic functionality. Securing device throughout the life cycle of the meter	-
M29	Remote update of cryptographic credentials	D
M30	Compliance of used algorithms with cryptographic requirements	-

A: Avalanche, D: DATEL, F: frequency analyzer, K: Kali Linux; ^1^ arpspoof, ^2^ Greenbone, ^3^ hping3, ^4^ nmap, ^5^ tcpreplay, ^6^ Wireshark.

## Data Availability

The authors will not publish the data online.

## References

[1] Zaraket C., Dogas I., Kalyvas D., Papageorgas P., Aillerie M., Agavanakis K. (2022). Open Source LoRaWAN Telemetry Test Bench for Smart Grid—A DLMS/COSEM Implementation Case Study. AIP Conf. Proc..

[2] (2020). Electricity metering decree, Act No. 359/2020 Coll. https://eur-lex.europa.eu/legal-content/CS/TXT/PDF/?uri=NIM:202100180.

[3] (2022). European Commission. NIS2 Directive. http://data.europa.eu/eli/dir/2022/2555/oj.

[4] (2014). Zákon 181/2014 Sb. o Kybernetické Bezpečnosti a o Změně Souvisejících Zákonů (Zákon o Kybernetické Bezpečnosti). https://www.govcert.cz/download/kii-vis/ZKB_uplne_zneni.pdf.

[5] (2019). SM-301-2019: Security Requirements for Procuring Smart Meters and Data Concentrators. Technical Report, ENCS. https://encs.eu/resource/sm-301-2019-security-requirements-for-procuring-smart-meters-and-data-concentrators/.

[6] Pudi S., Lagineni M., JaganMohan K., Kumar R., Bindhumadhava B. (2021). Secure DLMS/COSEM communication for Next Generation Advanced Metering Infrastructure. Asian J. Converg. Technol..

[7] Abdalzaher M.S., Fouda M.M., Emran A., Fadlullah Z.M., Ibrahem M.I. (2023). A Survey on Key Management and Authentication Approaches in Smart Metering Systems. Energies.

[8] Luring N., Szameitat D., Hoffmann S., Bumiller G. Analysis of security features in DLMS/COSEM: Vulnerabilities and countermeasures. Proceedings of the 2018 IEEE Power & Energy Society Innovative Smart Grid Technologies Conference (ISGT).

[9] Awad A.I., Shokry M., Khalaf A.A.M., Abd-Ellah M.K. (2023). Assessment of potential security risks in advanced metering infrastructure using the OCTAVE Allegro approach. Comput. Electr. Eng..

[10] Ur-Rehman O., Zivic N., Ruland C. Security issues in smart metering systems. Proceedings of the 2015 IEEE International Conference on Smart Energy Grid Engineering (SEGE).

[11] Bačnar D., Leytner L., Prenc R., Jardas V., Lerga J. On Security and Privacy In Smart Metering Systems. Proceedings of the 2022 7th International Conference on Smart and Sustainable Technologies (SpliTech).

[12] Sun C.C., Sebastian Cardenas D.J., Hahn A., Liu C.C. (2021). Intrusion Detection for Cybersecurity of Smart Meters. IEEE Trans. Smart Grid.

[13] Fehér M., Yazdani N., Aranha D.F., Lucani D.E., Hansen M.T., Vester F.E. Side Channel Security of Smart Meter Data Compression Techniques. Proceedings of the 2020 IEEE International Conference on Communications, Control, and Computing Technologies for Smart Grids (SmartGridComm).

[14] Prateek K., Maity S., Amin R. (2023). An Unconditionally Secured Privacy-Preserving Authentication Scheme for Smart Metering Infrastructure in Smart Grid. IEEE Trans. Netw. Sci. Eng..

[15] Halle P.D., Shiyamala S. (2022). Secure advance metering infrastructure protocol for smart grid power system enabled by the Internet of Things. Microprocess. Microsyst..

[16] Ju S.H., Seo H.S. (2018). Design key management system for DLMS/COSEM standardbased smart metering. Int. J. Eng. Technol..

[17] Kumar V., Hussain M. Secure communication for advance metering infrastructure in smart grid. Proceedings of the 2014 Annual IEEE India Conference (INDICON).

[18] Lieskovan T., Hajny J. Security of Smart Grid Networks in the Cyber Ranges. ARES22. Proceedings of the 17th International Conference on Availability, Reliability and Security.

[19] Xiaobing L., Wei C., Feng Z., Bin X., Zhiqiang S. Design of a security smart meter software testing cloud service system. Proceedings of the 2016 IEEE Information Technology, Networking, Electronic and Automation Control Conference.

[20] Goyal H., Purohit A. Landis+Gyr. Smart Meter Validation Test Bench. https://www.ni.com/cs-cz/innovations/case-studies/19/smart-meter-validation-test-bench.html.

[21] Leping Z., Baoshuai W., Shanshan H., Yi L., Yi P., Zhanhe W. (2019). Research on Key Test Methods of the Smart Meter Software Based on Failure Modes. J. Phy.: Conf. Ser..

[22] Janiga P., Liska M., Volcko V., Pilat B. Testing system for smart meters. Proceedings of the 2015 16th International Scientific Conference on Electric Power Engineering (EPE).

[23] Mendes H., Medeiros I., Neves N. Validating and Securing DLMS/COSEM Implementations with the ValiDLMS Framework. Proceedings of the 2018 48th Annual IEEE/IFIP International Conference on Dependable Systems and Networks Workshops (DSN-W).

[24] Marah R., Gabassi I.E., Larioui S., Yatimi H. Security of Smart Grid Management of Smart Meter Protection. Proceedings of the 2020 1st International Conference on Innovative Research in Applied Science, Engineering and Technology (IRASET).

[25] Mlynek P., Misurec J., Silhavy P., Fujdiak R., Slacik J., Hasirci Z. (2019). Simulation of Achievable Data Rates of Broadband Power Line Communication for Smart Metering. Appl. Sci..

[26] Mikulasek M., Dvorak R., Stusek M., Masek P., Mozny R., Mlynek P., Hosek J. NB-IoT vs LTE Cat M1: Demystifying Performance Differences under Varying Radio Conditions. Proceedings of the 2022 14th International Congress on Ultra Modern Telecommunications and Control Systems and Workshops (ICUMT).

[27] European Commission (2009). Mandate M441 for Smart Meters. https://energy.ec.europa.eu/mandate-m441-smart-meters-march-2009_en.

[28] Smart Meters Coordination Group (2016). SM-CG: Smart Grids and Meters. Technical Report, CEN, CENELEC, ETSI. https://www.cencenelec.eu/areas-of-work/cen-cenelec-topics/smart-grids-and-meters/smart-meters/.

[29] Smart Meters Coordination Group (2019). Protection Profile for Smart Meter Minimum Security Requirements. Technical Report, CEN, CENELEC, ETSI. https://www.esmig.eu/wp-content/uploads/2022/01/Protection-Profile-for-Smart-Meters.pdf.

[30] Smart Meters Coordination Group (2016). Minimum Security Requirements for AMI Components. Technical Report, CEN, CENELEC, ETSI. https://www.cencenelec.eu/media/CEN-CENELEC/AreasOfWork/CEN-CENELEC_Topics/SmartGridsandMeters/SmartMeters/smcg_sec0109.pdf.

[31] (2015). ETSI TR 103 118: Machine-to-Machine Communications (M2M); Smart Energy Infrastructures Security; Review of Existing Security Measures and Convergence Investigations. Technical Report, ETSI. https://www.etsi.org/deliver/etsi_tr/103100_103199/103118/01.01.01_60/tr_103118v010101p.pdf.

[32] (2020). ETSI TR 103 644: Observations from the SUCCESS Project Regarding Smart Meter Security. Technical Report, ETSI. https://www.etsi.org/deliver/etsi_tr/103600_103699/103644/01.02.01_60/tr_103644v010201p.pdf.

[33] Leszczyna R. (2019). Cybersecurity in the Electricity Sector: Managing Critical Infrastructure.

[34] Cervingi G., Larouche P. (2014). Regulating smart metering in Europe: Technological, Economic and Legal Challenges. Technical Report, CERRE. https://cerre.eu/publications/regulating-smart-metering-europe-technological-economic-and-legal-challenges/.

[35] European Commission (2020). Critical Infrastructure and Cybersecurity. https://energy.ec.europa.eu/topics/energy-security/critical-infrastructure-and-cybersecurity_en.

[36] (2020). Green Book: DLMS/COSEM Architecture and Protocols.

[37] (2020). Blue Book: COSEM Interface Classes and OBIS Object Identification System.

[38] Lieskovan T., Hajny J., Cika P. Smart Grid Security: Survey and Challenges. Proceedings of the 11th International Congress on Ultra Modern Telecommunications and Control Systems and Workshops (ICUMT).

[39] Barker E., Chen L., Keller S., Roginsky A., Vassilev A., Davis R. (2018). Recommendation for Pair-Wise Key-Establishment Schemes Using Discrete Logarithm Cryptography.

[40] Barker E. (2020). Recommendation for Key Management: Part 1—General.

[41] ECRYPT–CSA (2018). Algorithms, Key Size and Protocols Report.

[42] Smart N., European Union Agency for Cybersecurity (2014). Algorithms, Key Size and Parameters: Report—2014.

[43] NÚKIB (2022). Minimální Požadavky na Kryptografické Algoritmy. https://www.nukib.cz/download/publikace/podpurne_materialy/Kryptograficke_prostredky_doporuceni_v2.0.pdf.

[44] Barker E., Roginsky A., Davis R. (2017). Recommendation for Cryptographic Key Generation.

[45] (2020). NAP SG–Safety Requirements for Smart Meters and Related Infrastructure. https://www.mpo.cz/assets/cz/energetika/strategicke-a-koncepcni-dokumenty/narodni-akcni-plan-pro-chytre-site/2020/5/Vytah-studie-NAP-SG-kyberneticka-bezpecnost.pdf.

[46] Kohout D., Lieskovan T., Masek P., Slacik J., Mlynek P. (2022). Project-Testing the Cyber Security of Smart Electricity Meters 1. https://www.vut.cz/en/rad/projects/detail/34928.

[47] Kohout D., Lieskovan T., Masek P., Slacik J., Mlynek P. (2022). Project-Testing the Cyber Security of Smart Electricity Meters 2. https://www.vut.cz/en/rad/projects/detail/34927.

[48] (2016). National Action Plan for Smart Grids (NAP SG). https://www.mpo.cz/en/energy/electricity/national-action-plan-for-smart-grids-nap-sg--221572/.

[49] Gurux DLMS Library. https://www.gurux.fi/.

[50] Kohout D., Mlýnek P. Testing Smart Meters with Custom Application. Proceedings of the 2022 IEEE International Carnahan Conference on Security Technology (ICCST).

[51] Mlýnek P., Mašek P., Fujdiak R., Sláčik J. Roll-out chytrých elektroměru s NB-IoT/LTE Cat M–reálné zkušenosti. Proceedings of the Sborník konference ČK CIRED 2022.

